# Burden of gram-positive bacteria from urinary tract infection: *A four-year retrospective study in a tertiary health setting in southern Saudi Arabia*

**DOI:** 10.1371/journal.pone.0328042

**Published:** 2025-08-01

**Authors:** Abdullah M. Alkahtani, Abdulah J. Alqahtani, Abdulaziz Alamri, Mohammed Mushabab Assiry, Mona Elfaki, Yahya Mohammed Shabi, Mohamed E. Hamid

**Affiliations:** 1 Department of Microbiology and Clinical Parasitology, College of Medicine, King Khalid University, Abha, Saudi Arabia; 2 Departments of Surgery and Medical Education, College of Medicine, King Khalid University, Abha, Saudi Arabia; 3 Main Laboratory, Aseer Central Hospital, Ministry of Health, Abha, Saudi Arabia; Ahvaz Jundishapur University of Medical Sciences: Ahvaz Jondishapour University of Medical Sciences, IRAN, ISLAMIC REPUBLIC OF

## Abstract

**Background:**

Antibiotic resistance is a worldwide problem causing significant health-related and economic losses. Gram-positive causes of urinary tract infections (UTIs) are usually underestimated or overlooked by physicians.

**Aim:**

To examine the prevalence of antibiotic resistance among major gram-positive bacteria from UTIs in a tertiary care health hospital in southern Saudi Arabia.

**Method:**

A cross-sectional retrospective study was done in a tertiary health setting in southern Saudi Arabia between 2019 and 2022, to identify the major gram-positive bacteria and antibiotic resistance. Data were collected from the hospital records and was analyzed using the SPSS statistical package.

**Results:**

The most common gram-positive species were *Enterococcus faecalis* (44.7%), *Staphylococcus aureus* (15.1%), and *Enterococcus faecium* (12.9%), beta-hemolytic streptococci (8.4%), and methicillin-resistant *Staphylococcus aureus (MRSA)* (1.8%). The 1540 isolates showed an overall susceptibility of 71.0%, compared to a resistance of 29.0%. The most resistance was among *Enterococcus faecium* (54.5%), *Enterococcus gallinarum* (42.4%), *Enterococcus faecalis* (34.3%), and MRSA (27.2%). The most common resistance was to erythromycin (75.7%), followed by cefotaxime (73.9%), tetracycline (70.5%), ciprofloxacin (54.3%), and Synercid (53.6%). The prediction model indicates an increase in the prevalence of resistance in MRSA and, to a lesser extent, with *E. faecalis*, *E. faecium*, and beta-hemolytic streptococci.

**Conclusions:**

*Enterococcus faecalis* was the predominant gram-positive species, surpassing *Staphylococcus aureus*. Almost remarkable resistance was observed to most of the antibiotics that are frequently used in the study area, mainly erythromycin, cefotaxime, and tetracycline. Performing continuous monitoring of drug susceptibility may help with the empirical treatment of bacterial agents in the region.

## Introduction

The development of antibiotic resistance has become a serious worldwide health issue that makes treating infectious infections effectively extremely difficult. The abuse and misuse of antibiotics, inadequate infection control procedures, and the dearth of newly developed medications are some of the reasons contributing to the emergence of antibiotic-resistant bacteria. Globally, this problem causes significant health and financial damage [1]. Microbial resistance to antibiotics is a global health concern. Resistance mechanisms include β-lactamase production, efflux pumps or target site modifications leading to multidrug resistance bacteria emergence [2–4].

Information on the existence of the causative microorganisms and their susceptibility to commonly used antibiotics are essential to enhance therapeutic outcome [5–7]. Antimicrobial resistance and urinary tract infections continue to be the principal issues, bearing a heavy social and health cost, especially in developing nations. *Escherichia coli* is the main Gram-negative bacteria that typically causes this infection. Repeated reports of failure of empirical treatment in UTIs and reports of gram-positive bacteria as an important cause of UTIs evoked us to check the rates of infection and antimicrobial resistance pattern in Saudi Arabia [8].

Urinary tract infections (UTIs) are one of the most prevalent bacterial illnesses affecting millions of people worldwide [9]. Although there are many several types of bacteria that can cause UTIs, gram-negative bacteria, namely *E. coli* is the utmost common cause. Nonetheless, gram-positive bacteria have also been recognized as significant contributors to the burden of UTIs, comprising *Enterococcus* and *Staphylococcus* species [9–11].

Globally, UTIs placed amongst the most widespread bacterial infections, affecting approximately 150 million persons each year [9]. Gram-negative bacteria as *E. coli* and Gram-positive organisms as *Staphylococcus saprophyticus* are the main causes of UTIs, which can develop in the urinary tract, comprising the urethra, bladder, ureters, and kidneys [12]. The burden of UTIs is substantial, with women being mostly affected with up to a 50% risk of acquiring a UTI [13]. Recurring UTIs are also common complaint, with 20–30% of women facing a repeat infection within 6 months of the original incident [14]. A leading case of the significant financial burden of healthcare for UTIs is the about $2.8 billion that are encountered each year in the United States alone [9].

In Saudi Arabia, numerous reports have investigated the epidemiology of UTIs, with an emphasis on the etiology and antimicrobial resistance patterns [15–17]. These studies have underlined the increasing prevalence of gram-positive bacteria as causative agents of UTIs in the region. For example, a retrospective study conducted in Riyadh, Saudi Arabia, found that *Enterococcus* spp. accounted for up to 20% of UTI isolates [15]. The growing concern in Saudi Arabia about the event of antibiotic resistance, mainly to regularly administered drugs as trimethoprim-sulfamethoxazole and fluoroquinolones, is important [18,19]. High incidence of resistance was reported in Saudi Arabia for *Staphylococcus aureus*, 100% for cefazolin, 90.5% for fosfomycin, and 94% for fusidic acid; and for *Enterococcus species*, 97.3% for linezolid, 93% for vancomycin and 80.9% for nitrofurantoin [20].

The burden of Gram-positive bacteria in UTIs in the Aseer region of Saudi Arabia has received little attention. A previous study found that *E. coli* was the predominant pathogen; Gram-positive bacteria like *Staphylococcus saprophyticus* and *Enterococcus* species were also important pathogens [21] This pattern in Aseer region is in harmony with data from Saudi Arabia. Alzohairy and Khadri [22] stated that Gram-positive cocci represented 30–40% of UTI isolates countrywide, draw attention to the increased awareness and suitable management policies. A study from Aseer region found that the majority of the uro-pathogens revealed resistance to widely used antibiotics [21]. In contrast, among all uro-pathogens, vancomycin, daptomycin, and linezolid revealed the least resistance. Given their efficiency against resistant uropathogens, the data suggest that these antibiotics might be considered for empirical therapy of UTIs. Related research suggested the use of fosfomycin, cefoxitin, nitrofurantoin, and amoxicillin/clavulanate as the preferred first-line treatment options for UTIs. This recommendation is based on the relatively high in vitro activity of these antibiotics against the major bacterial causes of UTIs [23]. For more efficient UTI treatment, the researchers advise updating guidelines to include this latest antibiotic susceptibility data.

The purpose of this study was to look into the frequency of antibiotic resistance in common gram-positive bacteria that cause UTIs at a large southern Saudi Arabian tertiary care hospital.

## Materials and methods

### Study design

This study used a retrospective, cross-sectional design. The research investigated culture and antimicrobial susceptibility information gathered from patients (n = 1540) during 2019 and 2022 in a southern Saudi Arabian tertiary care health setting. Data was accessed on 20^th^ of January 2025. All patient records presented with UTIs as the main complaint and had complete records were recruited for this study without age, gender or severity restrictions.

### Data collection

The research identified the main urinary tract infection (UTI) gram-positive bacteria and analyzed the frequency of antibiotic resistance. Results of culture and antibiotic sensitivity of the bacterial isolates were collected from the record of the microbiology unit for the study period.

### Ethical consideration

The data collection was done after obtaining official permission from the King Khalid University institutional review board (ECME#2025-103). Data were collected anonymously. No data can reveal the identity of individual participants during or after data collection were accessed. Each patient submitted an informed consent to Aseer Central Hospital allowing the use of anonymous data for further analysis by Ministry of Health or other collaborators to help improving medical practice in Saudi Arabia.

### Clinical specimens and bacterial isolates

A specimen of midstream urine was collected and delivered to the microbiology lab for culture in a sterile collection tube. Before any samples were processed in the lab and cultured, they were all kept in the refrigerator. A loop full from each specimen was streaked onto MacConkey agar and blood agar plates, plates were incubated aerobically at 37°C for 24–48 hours.

Culture was considered positive by the presence of visible colony growth. Initially, a few morphological traits were used to identify the bacterial species, including Gram stain, culture, and biochemical tests then verified using the automatic Vitek microbial identification system in line with the guidelines provided by the manufacturer (BioMérieux SA, Marcy, France).

### Antimicrobial susceptibility tests

The resistance and sensitivity profiles for the isolated microbial species were determined using the automated Vitek system, in accordance with the manufacturer’s protocol (BioMérieux). We have examined 24 agents (plus a few others that are used less frequently). The five agents that have been used mostly are Synercid, ciprofloxacin, tetracycline, cefotaxime, and erythromycin.

### Data analysis

Using the statistical software program SPSS, the collected data was entered, inspected and analyzed. Descriptive statistics were computed to give an overview of the data. A bivariate logistic regression model was utilized to examine the association between the predictor variables, the organisms, and the antimicrobial drugs, as a result, the odds ratios and associated p-values will be estimated.

## Results

### The distribution of Gram-positive bacteria based on multiple parameters in urinary tract infections

**Fig 1:**
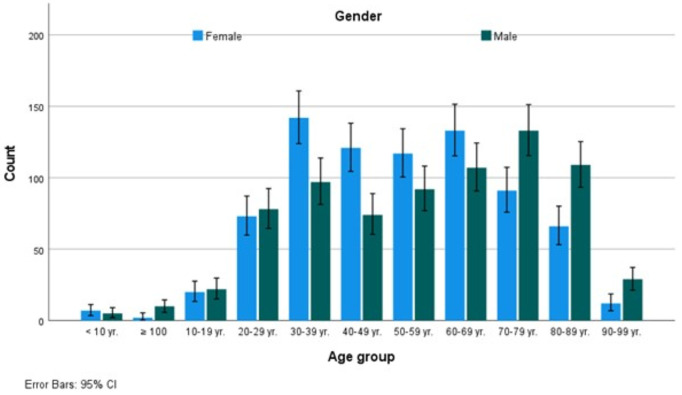
Age-specific distribution of gram-positive bacteria that cause urinary tract infections in females and males. Bars represent the standard error.

Table 1 provides an overview of the findings of our investigation about the gram-positive bacteria isolated from urinary tract infections. It shows the distribution according to different variables. The distribution of gram-positive bacteria in four years showed a consistent prevalence (p > 0.05), similarly, gender has shown a significant difference, with females at 784 (50.9%) and males at 756 (49.1%). Age groups have shown a tendency to increase in the Middle Ages, from 20–29 years old to 80–89 years old (Fig 1). The source of samples, and hence the infection, was mainly OPD, accounting for 1043 (67.7%) compared to 456 (29.6%) (p < 0.05).

**Table 1:** Distribution of gram-positive bacteria isolated from urinary tract infections according to year, gender, age group, and source of sample.

Criteria		*Total*		*Beta-hemolytic streptococci*		*Enterococcus casseliflavus*		*Enterococcus faecalis*		*Enterococcus faecium*		*Enterococcus gallinarum*		*Methicillin-resistant Staphylococcus aureus*		*Staphylococcus aureus*		*Streptococcus* sp.		*Streptococcus agalactiae*		*Streptococcus bovis*	
		N	%	N	%	N	%	N	%	N	%	N	%	N	%	N	%	N	%	N	%	N	%
Year	2019	476	30.9	53	41.1%	1	100.0%	201	29.2%	53	26.8%	1	33.3%	14	50.0%	51	22.0%	32	34.8%	70	41.7%	0	0.0%
	2020	314	20.4	30	23.3%	0	0.0%	131	19.0%	44	22.2%	0	0.0%	9	32.1%	42	18.1%	49	53.3%	9	5.4%	0	0.0%
	2021	443	28.8	40	31.0%	0	0.0%	217	31.5%	67	33.8%	1	33.3%	0	0.0%	77	33.2%	11	12.0%	29	17.3%	1	100.0%
	2022	307	19.9	6	4.7%	0	0.0%	139	20.2%	34	17.2%	1	33.3%	5	17.9%	62	26.7%	0	0.0%	60	35.7%	0	0.0%
Gender	Female	784	50.9	84	65.1%	0	0.0%	332	48.3%	105	53.0%	1	33.3%	10	35.7%	85	36.6%	65	70.7%	101	60.1%	1	100.0%
	Male	756	49.1	45	34.9%	1	100.0%	356	51.7%	93	47.0%	2	66.7%	18	64.3%	147	63.4%	27	29.3%	67	39.9%	0	0.0%
Age group	< 10 yr.	12	0.8	1	0.8%	0	0.0%	6	0.9%	0	0.0%	0	0.0%	0	0.0%	4	1.7%	0	0.0%	1	0.6%	0	0.0%
	≥ 100	12	0.8	0	0.0%	0	0.0%	6	0.9%	3	1.5%	0	0.0%	1	3.6%	2	0.9%	0	0.0%	0	0.0%	0	0.0%
	10-19 yr.	42	2.7	5	3.9%	0	0.0%	12	1.7%	6	3.0%	0	0.0%	3	10.7%	9	3.9%	2	2.2%	5	3.0%	0	0.0%
	20-29 yr.	151	9.8	9	7.0%	1	100.0%	64	9.3%	20	10.1%	0	0.0%	3	10.7%	26	11.2%	16	17.4%	12	7.1%	0	0.0%
	30-39 yr.	239	15.5	25	19.4%	0	0.0%	107	15.6%	19	9.6%	0	0.0%	7	25.0%	36	15.5%	21	22.8%	24	14.3%	0	0.0%
	40-49 yr.	195	12.7	23	17.8%	0	0.0%	75	10.9%	15	7.6%	0	0.0%	2	7.1%	26	11.2%	18	19.6%	36	21.4%	0	0.0%
	50-59 yr.	209	13.6	25	19.4%	0	0.0%	75	10.9%	23	11.6%	2	66.7%	3	10.7%	35	15.1%	11	12.0%	35	20.8%	0	0.0%
	60-69 yr.	240	15.6	23	17.8%	0	0.0%	89	12.9%	38	19.2%	0	0.0%	4	14.3%	35	15.1%	16	17.4%	34	20.2%	1	100.0%
	70-79 yr.	224	14.5	12	9.3%	0	0.0%	123	17.9%	39	19.7%	0	0.0%	2	7.1%	25	10.8%	7	7.6%	16	9.5%	0	0.0%
	80-89 yr.	175	11.4	4	3.1%	0	0.0%	109	15.8%	24	12.1%	1	33.3%	3	10.7%	30	12.9%	1	1.1%	3	1.8%	0	0.0%
	90-99 yr.	41	2.7	2	1.6%	0	0.0%	22	3.2%	11	5.6%	0	0.0%	0	0.0%	4	1.7%	0	0.0%	2	1.2%	0	0.0%
Source	EMR	9	0.6	1	0.8%	0	0.0%	5	0.7%	0	0.0%	0	0.0%	0	0.0%	2	0.9%	1	1.1%	0	0.0%	0	0.0%
	EXT	32	2.1	5	3.9%	0	0.0%	12	1.7%	2	1.0%	0	0.0%	1	3.6%	4	1.7%	5	5.4%	3	1.8%	0	0.0%
	INP	456	29.6	11	8.5%	0	0.0%	232	33.7%	133	67.2%	1	33.3%	10	35.7%	51	22.0%	7	7.6%	11	6.5%	0	0.0%
	OPD	1043	67.7	112	86.8%	1	100.0%	439	63.8%	63	31.8%	2	66.7%	17	60.7%	175	75.4%	79	85.9%	154	91.7%	1	100.0%

### Prevalence of gram-positive bacteria according to criteria

The prevalence over the four years showed persistent patterns (p > 0.05) effecting gender, age groups, and whether the source of the sample whether an outpatient department or inpatient.

### Dominant gram-positives species

**Fig 2:**
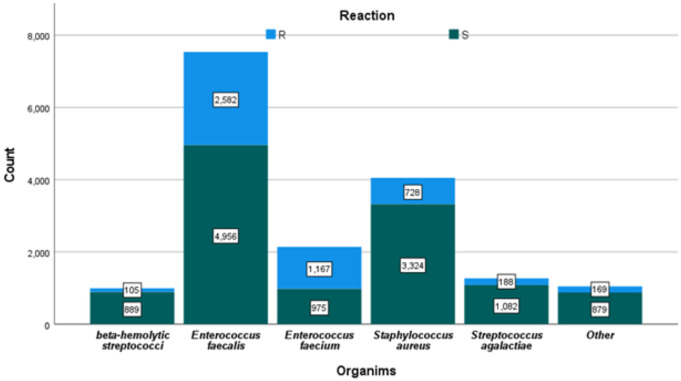
Major gram-positive bacterial counts and their corresponding susceptibility and resistance numbers (S = sensitive and R = Resistant).

The dominant gram-positives species *Enterococcus faecalis* (44.7%), *Staphylococcus aureus* (15.1%), *Enterococcus faecium* (12.9%), *Streptococcus agalactiae* (10.9%), beta-hemolytic streptococci (8.4%), *Streptococcus* sp. (6.0%), Methicillin-resistant *Staphylococcus* aureus (1.8%), *Enterococcus gallinarum* (0.2%), *Enterococcus casseliflavus* (0.1%), and *Streptococcus bovis* (0.1%) (Fig 2).

### Common resistance to antibiotics

The susceptibility of the studied bacteria to all tested antimicrobial agents is shown in Table 2. The 1540 isolates showed an overall susceptibility of 71.0%, compared to a resistance of 29.0%. The most common resistance was to erythromycin (75.7%), followed by cefotaxime (73.9%), tetracycline (70.5%), ciprofloxacin (54.3%), and Synercid (53.6%). The remaining antimicrobials revealed resistance rates of less than 50% (Table 2).

**Table 2:** Susceptibility of gram-positive bacteria to antimicrobial agents.

		*Beta-hemolytic streptococci*	*Enterococcus casseliflavus*	*Enterococcus faecalis*	*Enterococcus faecium*	*Enterococcus gallinarum*	*Methicillin-resistant Staphylococcus aureus*	*Staphylococcus aureus*	*Streptococcus* sp.	*Streptococcus agalactiae*	*Streptococcus bovis*
		Column N %	Column N %	Column N %	Column N %	Column N %	Column N %	Column N %	Column N %	Column N %	Column N %
**Amox/ K Clav**	R	0.0%	0.0%	0.0%	0.0%	0.0%	100%	6.9%	0.0%	0.0%	0.0%
	S	100.0%	0.0%	0.0%	0.0%	0.0%	0.0%	93.1%	0.0%	0.0%	0.0%
**Fosfomycin**	R	0.0%	0.0%	0.0%	0.0%	0.0%	9.5%	6.7%	0.0%	0.0%	0.0%
	S	0.0%	0.0%	0.0%	0.0%	0.0%	90.5%	93.3%	0.0%	0.0%	0.0%
**Nitrofurantoin**	R	0.0%	0.0%	5.6%	43.2%	0.0%	4.8%	1.0%	0.0%	2.3%	0.0%
	S	100.0%	100.0%	94.4%	56.8%	0.0%	95.2%	99.0%	100.0%	97.7%	0.0%
**Trimeth/ Sulfa**	R	0.0%	0.0%	93.3%	100.0%	0.0%	21.4%	10.2%	0.0%	0.0%	0.0%
	S	0.0%	0.0%	6.7%	0.0%	0.0%	78.6%	89.8%	0.0%	0.0%	0.0%
**Amikacin**	R	0.0%	0.0%	0.0%	0.0%	0.0%	0.0%	50.0%	0.0%	0.0%	0.0%
	S	0.0%	0.0%	100.0%	0.0%	0.0%	0.0%	50.0%	0.0%	0.0%	0.0%
**Amoxicillin**	R	14.3%	0.0%	0.0%	0.0%	0.0%	0.0%	0.0%	0.0%	0.0%	0.0%
	S	85.7%	0.0%	100.0%	100.0%	0.0%	0.0%	0.0%	100.0%	100.0%	0.0%
**Ampicillin**	R	0.0%	0.0%	11.4%	78.0%	100.0%	100.0%	65.0%	0.0%	0.0%	0.0%
	S	100.0%	100.0%	88.6%	22.0%	0.0%	0.0%	35.0%	100.0%	100.0%	100.0%
**Azithromycin**	R	0.0%	0.0%	0.0%	0.0%	0.0%	56.3%	37.3%	0.0%	0.0%	0.0%
	S	0.0%	0.0%	0.0%	0.0%	0.0%	43.8%	62.7%	0.0%	0.0%	0.0%
**Cefepime**	R	0.0%	0.0%	0.0%	0.0%	0.0%	0.0%	0.0%	0.0%	0.0%	0.0%
	S	0.0%	0.0%	0.0%	0.0%	0.0%	0.0%	0.0%	100.0%	100.0%	0.0%
**Cefotaxime**	R	0.0%	0.0%	100.0%	100.0%	0.0%	0.0%	0.0%	0.0%	0.0%	0.0%
	S	0.0%	0.0%	0.0%	0.0%	0.0%	0.0%	100.0%	100.0%	100.0%	0.0%
**Cefoxitin**	R	0.0%	0.0%	0.0%	0.0%	0.0%	0.0%	40.0%	0.0%	0.0%	0.0%
	S	0.0%	0.0%	0.0%	0.0%	0.0%	0.0%	60.0%	0.0%	0.0%	0.0%
**Cephalothin**	R	0.0%	0.0%	0.0%	0.0%	0.0%	100.0%	0.0%	0.0%	0.0%	0.0%
	S	0.0%	0.0%	0.0%	0.0%	0.0%	0.0%	100.0%	0.0%	0.0%	0.0%
**Chloramphenicol**	R	0.0%	0.0%	13.8%	25.0%	0.0%	0.0%	0.0%	0.0%	0.0%	0.0%
	S	0.0%	0.0%	86.2%	75.0%	0.0%	100.0%	100.0%	0.0%	0.0%	0.0%
**Ciprofloxacin**	R	0.0%	0.0%	53.9%	83.6%	100.0%	45.0%	34.6%	0.0%	0.0%	0.0%
	S	100.0%	100.0%	46.1%	16.4%	0.0%	55.0%	65.4%	0.0%	0.0%	0.0%
**Clarithromycin**	R	0.0%	0.0%	0.0%	0.0%	0.0%	33.3%	20.0%	0.0%	0.0%	0.0%
	S	0.0%	0.0%	0.0%	0.0%	0.0%	66.7%	80.0%	0.0%	0.0%	0.0%
**Clindamycin**	R	30.8%	0.0%	95.7%	100.0%	0.0%	22.2%	19.0%	16.7%	59.1%	0.0%
	S	69.2%	0.0%	4.3%	0.0%	0.0%	77.8%	81.0%	83.3%	40.9%	100.0%
**Daptomycin**	R	0.0%	0.0%	0.0%	0.0%	0.0%	0.0%	0.0%	0.0%	0.0%	0.0%
	S	100.0%	100.0%	100.0%	100.0%	100.0%	100.0%	100.0%	100.0%	100.0%	100.0%
**Erythromycin**	R	45.5%	0.0%	87.1%	90.1%	50.0%	50.0%	37.0%	50.0%	78.6%	0.0%
	S	54.5%	0.0%	12.9%	9.9%	50.0%	50.0%	63.0%	50.0%	21.4%	0.0%
**Fusidc Acid**	R	0.0%	0.0%	100.0%	0.0%	0.0%	28.6%	23.0%	0.0%	0.0%	0.0%
	S	0.0%	0.0%	0.0%	0.0%	0.0%	71.4%	77.0%	0.0%	0.0%	0.0%
**Gent.Synergy**	R	50.0%	0.0%	47.1%	45.9%	0.0%	0.0%	0.0%	0.0%	0.0%	0.0%
	S	50.0%	0.0%	52.9%	54.1%	100.0%	0.0%	0.0%	0.0%	0.0%	0.0%
**Gentamicin**	R	0.0%	0.0%	100.0%	100.0%	0.0%	7.4%	14.3%	0.0%	0.0%	0.0%
	S	0.0%	0.0%	0.0%	0.0%	0.0%	92.6%	85.7%	0.0%	0.0%	0.0%
**Imipenem**	R	0.0%	0.0%	0.0%	0.0%	0.0%	100.0%	7.6%	0.0%	0.0%	0.0%
	S	0.0%	0.0%	0.0%	0.0%	0.0%	0.0%	92.4%	0.0%	0.0%	0.0%
**Inducible Clindamycin**	R	0.0%	0.0%	0.0%	0.0%	0.0%	0.0%	100.0%	0.0%	0.0%	0.0%
	S	0.0%	0.0%	0.0%	0.0%	0.0%	0.0%	0.0%	0.0%	0.0%	0.0%
**Levofloxacin**	R	4.1%	0.0%	50.8%	84.7%	100.0%	46.4%	32.9%	9.1%	6.5%	0.0%
	S	95.9%	100.0%	49.2%	15.3%	0.0%	53.6%	67.1%	90.9%	93.5%	100.0%
**Linezolid**	R	0.0%	0.0%	2.9%	4.2%	0.0%	0.0%	4.0%	0.0%	0.0%	0.0%
	S	100.0%	100.0%	97.1%	95.8%	100.0%	100.0%	96.0%	100.0%	100.0%	100.0%
**Meropenem**	R	0.0%	0.0%	0.0%	0.0%	0.0%	0.0%	0.0%	0.0%	0.0%	0.0%
	S	100.0%	0.0%	0.0%	0.0%	0.0%	0.0%	0.0%	0.0%	100.0%	0.0%
**Moxifloxacin**	R	14.3%	0.0%	25.0%	0.0%	0.0%	44.4%	28.9%	0.0%	10.8%	0.0%
	S	85.7%	0.0%	75.0%	0.0%	0.0%	55.6%	71.1%	100.0%	89.2%	0.0%
**Mupirocin**	R	0.0%	0.0%	0.0%	0.0%	0.0%	5.9%	4.5%	0.0%	0.0%	0.0%
	S	0.0%	0.0%	0.0%	0.0%	0.0%	94.1%	95.5%	0.0%	0.0%	0.0%
**Netilmicin**	R	0.0%	0.0%	0.0%	0.0%	0.0%	0.0%	10.0%	0.0%	0.0%	0.0%
	S	0.0%	0.0%	0.0%	0.0%	0.0%	100.0%	90.0%	0.0%	0.0%	0.0%
**Norfloxacin**	R	0.0%	0.0%	33.3%	100.0%	0.0%	50.0%	0.0%	0.0%	0.0%	0.0%
	S	0.0%	0.0%	66.7%	0.0%	0.0%	50.0%	100.0%	0.0%	0.0%	0.0%
**Oxacillin**	R	0.0%	0.0%	0.0%	0.0%	0.0%	100.0%	41.6%	0.0%	0.0%	0.0%
	S	0.0%	0.0%	0.0%	0.0%	0.0%	0.0%	58.4%	0.0%	0.0%	0.0%
**Penicillin**	R	0.0%	0.0%	16.8%	81.5%	66.7%	100.0%	90.6%	0.0%	0.0%	0.0%
	S	100.0%	100.0%	83.2%	18.5%	33.3%	0.0%	9.4%	100.0%	100.0%	100.0%
**Rifampin**	R	0.0%	0.0%	27.8%	82.5%	0.0%	0.0%	3.8%	0.0%	0.0%	0.0%
	S	0.0%	100.0%	72.2%	17.5%	100.0%	100.0%	96.2%	0.0%	0.0%	0.0%
**Strep.Synergy**	R	0.0%	0.0%	50.0%	20.0%	0.0%	0.0%	0.0%	0.0%	0.0%	0.0%
	S	0.0%	0.0%	50.0%	80.0%	0.0%	0.0%	0.0%	0.0%	0.0%	0.0%
**Synercid**	R	0.0%	0.0%	92.6%	30.3%	0.0%	0.0%	6.2%	0.0%	2.9%	0.0%
	S	100.0%	0.0%	7.4%	69.7%	100.0%	100.0%	93.8%	100.0%	97.1%	100.0%
**Teicoplanin**	R	0.0%	0.0%	5.9%	42.7%	33.3%	3.6%	2.4%	0.0%	0.0%	0.0%
	S	100.0%	100.0%	94.1%	57.3%	66.7%	96.4%	97.6%	0.0%	0.0%	0.0%
**Tetracycline**	R	91.2%	0.0%	88.6%	60.5%	33.3%	25.0%	18.8%	100.0%	77.2%	100.0%
	S	8.8%	100.0%	11.4%	39.5%	66.7%	75.0%	81.2%	0.0%	22.8%	0.0%
**Tigecycline**	R	0.0%	0.0%	0.0%	0.0%	0.0%	0.0%	0.0%	0.0%	0.0%	0.0%
	S	100.0%	0.0%	100.0%	100.0%	100.0%	100.0%	100.0%	100.0%	100.0%	0.0%
**Tobramycin**	R	0.0%	0.0%	0.0%	0.0%	0.0%	14.3%	19.0%	0.0%	0.0%	0.0%
	S	0.0%	0.0%	0.0%	0.0%	0.0%	85.7%	81.0%	0.0%	0.0%	0.0%
**Vancomycin**	R	0.8%	0.0%	2.7%	37.9%	33.3%	0.0%	0.0%	0.0%	0.0%	0.0%
	S	99.2%	100.0%	97.3%	62.1%	66.7%	100.0%	100.0%	100.0%	100.0%	100.0%
**Without antibiotics**	R	0.0%	0.0%	0.0%	0.0%	0.0%	0.0%	0.0%	0.0%	0.0%	0.0%
	S	100.0%	0.0%	100.0%	0.0%	0.0%	0.0%	100.0%	0.0%	0.0%	0.0%

**Fig 3:**
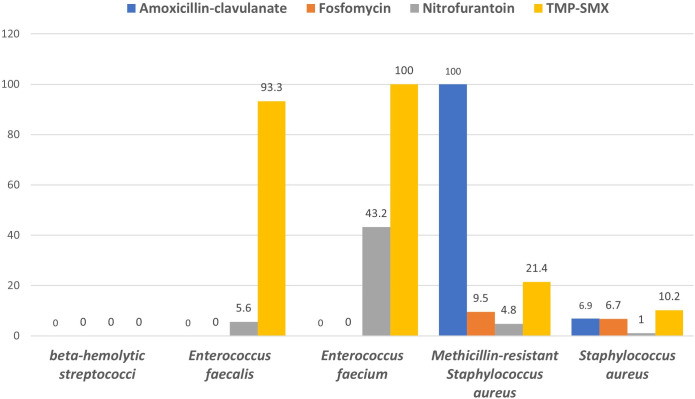
Resistance rate (%) of major gram-positive bacteria to commonly used antimicrobial agents for uncomplicated UTIs.

Resistance rates of major gram-positive bacteria to commonly used as treatment agents for uncomplicated UTIs are shown in Fig 3.

**Fig 4:**
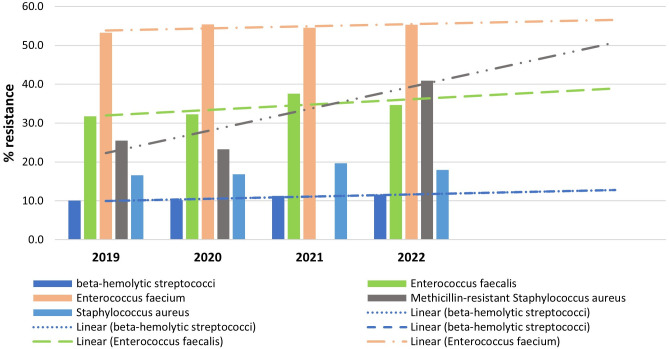
Overall resistance of the main gram-positive bacteria to antibiotics and orientation of the prediction lines.

The direction of the prediction lines and the general antibiotic resistance of the major gram-positive bacteria are displayed in Fig 4. This prediction model indicates an increasing in the prevalence of resistance in MRSA and to a lesser extent with E. faecalis, E. faecium, and least is beta-hemolytic streptococci.

### Predictive model for infectivity by

Table 3 presents a predictive model for the infectivity of three urinary tract infection (UTI) organisms – *Enterococcus faecalis, Enterococcus faecium*, and GBS – based on a multiple logistic regression analysis of patient data from southern Saudi Arabia. The multivariate regression analysis did not detect any important risk factors associated with the causation and prevalence of UTI-causing organisms. The odds ratios (Exp(B) values) and their corresponding p-values did not present a statistically significant link between any of the demographic factors checked and the incidence of UTI-causing organisms.

**Table 3:** Predictive model for infectivity by the three major UTI organisms (*E. faecalis, E. faecium,* and GBS*)* recovered from patients in southern Saudi Arabia according to multiple logistic regression analysis.

Organims*	B	Std. Error	Wald	df	Sig.	Exp (B)	95% Confidence Interval for Exp(B)	
							Lower Bound	Upper Bound
**Beta-hemolytic streptococci**								
** Intercept**	28.893	1595.325	0.000	1	0.986			
** [Gender=1]**	−12.337	712.044	0.000	1	0.986	E	0.000	.b
** [Gender=2]**	0c			0				
** [Age=1]**	0.896	9400.602	0.000	1	1.000	2.450	0.000	.b
** [Age=2]**	−13.731	8649.744	0.000	1	0.999	E	0.000	.b
** [Age=3]**	1.397	4859.505	0.000	1	1.000	4.043	0.000	.b
** [Age=4]**	0.696	2876.406	0.000	1	1.000	2.007	0.000	.b
** [Age=5]**	1.412	2412.735	0.000	1	1.000	4.105	0.000	.b
** [Age=6]**	1.480	1.038	2.033	1	0.154	4.394	0.574	33.619
** [Age=7]**	1.446	1.036	1.949	1	0.163	4.247	0.557	32.361
** [Age=8]**	−13.924	1427.606	0.000	1	0.992	E	0.000	.b
** [Age=9]**	0.097	2364.515	0.000	1	1.000	1.102	0.000	.b
** [Age=10]**	−0.844	2588.550	0.000	1	1.000	0.430	0.000	.b
** [Age=11]**	0c			0				
** [Source=1]**	13.738	6284.871	0.000	1	0.998	E	0.000	.b
** [Source=2]**	1.401	4003.322	0.000	1	1.000	4.060	0.000	.b
** [Source=3]**	11.021	882.936	0.000	1	0.990	E	0.000	.b
** [Source=4]**	0c			0				
**Enterococcus faecalis**								
**Intercept**	31.168	1595.325	0.000	1	0.984			
** [Gender=1]**	−12.857	712.044	0.000	1	0.986	E	0.000	.b
** [Gender=2]**	0c			0				
** [Age=1]**	0.988	9400.602	0.000	1	1.000	2.685	0.000	.b
** [Age=2]**	−0.731	8601.793	0.000	1	1.000	0.482	0.000	.b
** [Age=3]**	0.092	4859.505	0.000	1	1.000	1.096	0.000	.b
** [Age=4]**	0.488	2876.406	0.000	1	1.000	1.630	0.000	.b
** [Age=5]**	0.713	2412.735	0.000	1	1.000	2.039	0.000	.b
** [Age=6]**	0.370	0.776	0.227	1	0.634	1.447	0.316	6.621
** [Age=7]**	0.339	0.775	0.192	1	0.661	1.404	0.307	6.411
** [Age=8]**	−14.799	1427.606	0.000	1	0.992	E	0.000	.b
** [Age=9]**	0.022	2364.515	0.000	1	1.000	1.022	0.000	.b
** [Age=10]**	0.005	2588.550	0.000	1	1.000	1.005	0.000	.b
** [Age=11]**	0c			0				
** [Source=1]**	13.995	6284.871	0.000	1	0.998	E	0.000	.b
** [Source=2]**	0.556	4003.322	0.000	1	1.000	1.744	0.000	.b
** [Source=3]**	12.608	882.936	0.000	1	0.989	E	0.000	.b
** [Source=4]**	0c			0				
**Enterococcus faecium**								
** Intercept**	29.663	1595.325	0.000	1	0.985			
** [Gender=1]**	−12.552	712.044	0.000	1	0.986	E	0.000	.b
** [Gender=2]**	0c			0				
** [Age=1]**	−13.092	9427.803	0.000	1	0.999	E	0.000	.b
** [Age=2]**	−0.592	8601.793	0.000	1	1.000	0.553	0.000	.b
** [Age=3]**	0.156	4859.505	0.000	1	1.000	1.169	0.000	.b
** [Age=4]**	0.096	2876.406	0.000	1	1.000	1.101	0.000	.b
** [Age=5]**	−0.321	2412.735	0.000	1	1.000	0.725	0.000	.b
** [Age=6]**	−0.729	0.866	0.708	1	0.400	0.482	0.088	2.634
** [Age=7]**	−0.156	0.850	0.034	1	0.855	0.856	0.162	4.527
** [Age=8]**	−14.997	1427.606	0.000	1	0.992	E	0.000	.b
** [Age=9]**	−0.591	2364.515	0.000	1	1.000	0.554	0.000	.b
** [Age=10]**	−0.979	2588.550	0.000	1	1.000	0.376	0.000	.b
** [Age=11]**	0c			0				
** [Source=1]**	0.638	6342.719	0.000	1	1.000	1.893	0.000	.b
** [Source=2]**	0.969	4003.322	0.000	1	1.000	2.636	0.000	.b
** [Source=3]**	14.038	882.936	0.000	1	0.987	E	0.000	.b
** [Source=4]**	0c			0				

a. The reference category is: *Streptococcus bovis*.

b. Floating point overflow occurred while computing this statistic. Its value is therefore set to system missing.

c. This parameter is set to zero because it is redundant.

E. The value of Ex (B) is very high.

## Discussion

The present study attracts attention to the high incidence and patterns of antibiotic resistance of *Staphylococcus aureus* and *Enterococcus faecalis* among the survived community. The result that *E. faecalis* be more than *S. aureus* is noteworthy because *E. faecalis* is known to be innately more resistant to a wide range of widely used antibiotics [24]. It is notably worrying to observe the high rates of resistance to antibiotics for example erythromycin, cefotaxime, and tetracycline. Their poorer susceptibility denotes that there are not much effective empirical treatment possibilities, even if they are often given antimicrobials. Our present prediction model likewise displays a worrying trend of increasing MRSA resistance, which makes treating these infections even more difficult. Significant resistance was also noticed in *Enterococcus faecium*, one more bacterium of clinical implication. This is important since infections caused by vancomycin-resistant *Enterococcus* (VRE) are hard to cure and have a bad prognosis [25].

The epidemiology of urinary tract infections (UTIs) in Saudi Arabia has revealed alarming results about antibiotic resistance that is in line our present finding and the worldwide trends [26]. Many studies have been done in the region which underlined the increasing occurrence of gram-positive bacteria, such as Enterococcus species, as causative agents of UTIs. For example, a retrospective analysis in Riyadh, Saudi Arabia observed that Enterococcus spp. accounted for up to 20% of UTI isolates [23]. The increasing global resistance and the trend towards gram-positive infections playing a larger role in UTIs highlight the need for improved surveillance and antimicrobial stewardship initiatives in Saudi Arabia and the Middle East as a whole. The growing prevalence of gram-positive bacteria, such as *Enterococcus*, as causative agents of UTIs in Saudi Arabia, along with the universal increase in antimicrobial resistance, underscores the need for improved surveillance and enhanced antimicrobial stewardship efforts in the area. In Jazan, the incidence of UTIs caused by bacteria resistant to antibiotics is notable. As stated by study from other districts, *E. coli* persists to be the common bacteria accountable for UTIs and has a seasonal display that requires further attention. Multi-drug-resistant organism comprised about 35% of documented cases, with ESBLs making up 30% of those cases [19].

Understanding the role of causative microorganisms and their antibiotic resistance patterns is crucial for developing effective treatments and improving patient outcomes. Continued research, surveillance, and comprehensive interventions are essential to address this growing public health threat [27]. The present study found that the dominant gram-positive bacteria isolated from urinary tract infections (UTIs) were Enterococcus faecalis, Staphylococcus aureus, and Enterococcus faecium. Flores-Mireles et al. [9] discussed the epidemiology of UTIs, including the common causative pathogens such as Enterococcus faecalis, Staphylococcus aureus, and Enterococcus faecium. Their findings are in line with our ranking Enterococcus faecalis as number one gram-positive bacteria in UTI causal agents. Other work provides supporting evidence for the conclusion that the dominant gram-positive bacteria isolated from urinary tract infections (UTIs) were Enterococcus faecalis, Staphylococcus aureus, and Enterococcus faecium [28]. Many other studies have reported that Enterococcus faecalis prevalence ranges from 16–26% [29–31].

The current study highlighted the rising problems with antibiotic resistance, especially with Enterococcus species, in the community under study in southern Saudi Arabia. According to the current results, erythromycin resistance was the most common antibiotic resistance next in decreasing order of prevalence was resistance to cefotaxime, tetracycline, ciprofloxacin, and Synercid. Less than 50% of the remaining antimicrobials showed signs of resistance. A study in 2023 examined antibiotic resistance trends in UTIs across the United States. The study found that resistance to commonly prescribed UTI antibiotics like trimethoprim-sulfamethoxazole and ciprofloxacin reached 25–30% nationwide by 2022. Worryingly, the researchers also identified increasing resistance to last-line therapies like nitrofurantoin and fosfomycin, reaching 10–15% in some regions [32].

Microorganisms are becoming increasingly resistant to antibiotics; this is a developing worldwide matter. This is produced by the abuse and misuse of antibiotics, which obliges bacteria to evolve and promotes the growth of resistant species [1]. The problem can be further aggravated as these resistant bacteria share their resistance make-ups through genetic exchange [33,34]. Thus, infections conveyed by microorganisms that have developed resistance become hard to treat. Patients will therefore demand further cost for medications, stay in the hospital longer, and have a greater possibility of complications and death. This increase in antibiotic resistance adding a substantial burden on international economies and healthcare systems [35].

The analysis’s findings in this study corroborate previous research in showing that UTI-causing organisms may not be principally determined by demographic factors. However, numerous demographic characters, such as age and sex, were linked to an increased incidence of UTIs, the influences were often trivial and unreliable across studies, as stated by a systematic study by Smith et al. [36]. Similarly, clinical and behavioral factors like sexual activity, antibiotic use, and underlying diseases were found to be greater predictors of UTI incidence than standard demographic characteristics [37].

These studies show up the complex, versatile etiology of UTIs. UTIs are caused by a dynamic communication between the pathogen, the host, and environmental factors that can vary from person to person [9].

The findings of prediction model in the current study showed rising resistance tendencies, this worrying and coherent with broader universal patterns of antimicrobial resistance, particularly for MRSA and to a lesser extent for *Enterococcus* species and beta-hemolytic streptococci. In the previous 20 years, reports have revealed that the occurrence of MRSA infections has risen in healthcare as well as in community settings [38,39]. The increasing resistance among beta-hemolytic streptococci, such as group A Streptococcus, has also been reported, further complicating treatment of severe invasive infections [40]. To fight these increases in resistance and assure inexpensive management of infections caused by these pathogens, continuing surveillance and the formation of state-of-the-art antimicrobial strategies will be important. With the use of this data, policymakers ought to urge the careful use of antibiotics to reduce the increase of resistant organisms and put in place strong mechanisms for infection prevention and control. Future research has a responsibility to investigate the causal means behind the registered resistance and create pioneering therapeutic strategies to combat the infections that are difficult to manage.

## Conclusions

This study highlights the importance of gram-positive bacteria as causative pathogens of UTIs and describes antibiotic resistance in southern Saudi Arabia. Increasing resistance in MRSA is a big concern. Continuous monitoring of antibiotic susceptibility is essential to guide empirical treatment. Follow-up on resistance is so important to guide antibiotics stewardship and lower the cost and patient’s hospitalization.
